# Future of myocardial infarction mortality in Iran: a scenario-based study

**DOI:** 10.1186/s41043-023-00356-8

**Published:** 2023-03-16

**Authors:** Gisoo Alizadeh, Kamal Gholipour, Maryam Kazemi Shishavan, Reza Dehnavieh, Salime Goharinejad, Morteza Arab-Zozani, Mohammad Farough Khosravi, Rahim Khodayari-Zarnaq

**Affiliations:** 1 Tabriz Health Services Management Research Center, School of Management and Medical Informatics, Tabriz University of Medical Sciences, Daneshgah Street, 5165665811, Tabriz, Iran; 2 Department of Family and Community Medicine, Tabriz University of Medical Sciences, Tabriz, Iran; 3 Health Services Management Research Center, Institute for Futures Studies in Health, Kerman University of Medical Sciences, Kerman, Iran; 4 Preventive Medicine and Public Health Research Center, Psychosocial Health Research Institute, Iran University of Medical Sciences, P.O. Box: 1449614535, Tehran, Iran; 5 Social Determinants of Health Research Center, Birjand University of Medical Sciences, Birjand, Iran; 6 Department of Health Economics and Management, School of Public Health Tehran University of Medical Sciences, Tehran, Iran

**Keywords:** Futures study, Myocardial infarction, Non-communicable diseases, Scenario development, Policy option

## Abstract

This study defines futures myocardial infarction landscapes and proposes a few policy options to reduce the burden of cardiovascular diseases using the scenario development method. We identified the effective drivers of myocardial infarction by reviewing the literature and completed the returned list with “experts” opinions. The results were classified using the STEEP (Social, Technological, Environmental, Economic, and Political) framework. We plotted the critical uncertainties in a two-dimensional ranking of “effect” and “uncertainty” levels. Eleven drivers with uncertainty and high potential impact were selected and categorized into three groups: Political Development, Access to health services, and Self-Care. Scenarios were developed, and 3 scenarios (optimistic, pessimistic, and possible) were selected based on scoring. For each scenario, policy options were formulated. Utilizing the capacity of Non-Governmental Organizations and charities and strengthening restrictive and punitive legislation was chosen as policy options for addressing possible scenarios. Building infrastructure and improving prevention services, designing and regenerating curative infrastructure were selected as optimal strategies for addressing issues related to the optimistic scenario. Strengthening restrictive and punitive legislation related to community health and population empowerment were proposed as critical policy options for health improvement regarding the pessimistic scenario. Increasing people’s participation, strengthening infrastructure and punitive policies can be effective in Myocardial infarction mortality prevention policies in Iran.

**Supplementary Information:**

The online version contains supplementary material available at 10.1186/s41043-023-00356-8.

## Introduction

Futures study is a scientific study in which scientists use a wide range of methods to analyze the past and the present and collect data to predict futures in the various fields of science and technology [1]. Recently, adopting futures studies for planning, decision-making, and policymaking has gained popularity in Iran [2]. There are various methods for conducting futures studies, the most famous of which are time series analysis, modeling, simulation, and scenario development [3]. Scenario development is commonly used in economics, studies of society, culture, and politics. Initially, this method is known as the primary tool used in information analysis.

Scientists use futures studies in health-related studies to explore various diseases. Admittedly, its benefits are indicated by a scoping review published in 2015. In this review, while authors were outlining the application of scenario development method in health-related studies, they marked the benefits of using this technique for health care planning and strategic decision-making in public health [4, 5]. The advantages of adopting the scenario development method are also evident in “The bioeconomy to 2030” project for the Organization for Economic Cooperation and Development countries. After depicting the developed scenarios in this project, final interests were supported using various policy options [6]. Also, in the study that it was published in 2007 to study the future of IS Organization in 2020. Four scenarios have been proposed based on differing assumptions about two drivers: the advances in the reliability of international telecommunications and the value placed on computerization in businesses and society [7]. Non-communicable diseases are important issues in the field of health that future studies can be used to improve prevention, treatment, and rehabilitation services. Due to the importance of non-communicable diseases, it is one of the fields that can be used in futures studies [8].

Non-communicable diseases are essential health issues, and futures studies can be used to improve prevention, treatment and rehabilitation [8]. This is because many non-communicable diseases, such as cardiovascular diseases, usually burden the working-age population and the elderly. Consequently, their overall consequences wreak their toll on almost everybody in the community and getting ready for the possible scenarios might have an underestimated benefits [9]. In this regard, indicated enough by bloom et al. between 2011 and 2030, diabetes, cancer, chronic lung disease, and cardiovascular disease will be accounted for about $30 trillion in economic loss [10], and the results of the modeling study showed that the burden of CVD would increase steeply in Iran over 2005–2025 [11]. Regretfully, about 80% of CVD deaths occur in low- and middle-income countries where they are more exposed to risk factors such as tobacco, which lead to CVDs and other non-communicable diseases [12]. However, the burden of non-communicable diseases is not restricted to just low- and middle-income countries; a study in Ireland shows that the prevalence of being overweight and obese will increase by 89% and 85% in men and women by 2030, respectively, leading to an increase in the prevalence of cardiovascular disease, cancer and diabetes type 2 [13]. Another study in China reported that 2.9 to 5.7 million deaths could be prevented by reducing risk factors for cardiovascular diseases until 2030 [13]. Overall, the evidence on the necessity of implementing effective interventions aiming to reduce cardiovascular disease attributed risk factors in forthcoming years is abundant, which urges and justifies assessing Iran’s possible future cardiovascular-related scenarios and developing appropriate policy options [14, 15]. In order to reduce the burden of cardiovascular diseases, setting strategies for treating and preventing non-communicable diseases has become the main focus of the World Health Organization’s (WHO) Joint Prevention Programs [16]. Furthermore, align with The United Nations(UN), WHO calls for a 25% reduction in mortality from non-communicable diseases in 30–70 years by 2025 [17]. This objective is expected to be achieved by the countries by setting nation-specific goals and action plans because different countries have different contexts and priorities and often have different healthcare provision and management systems [18]. Fortunately, Iran is among the first developing countries that pursued this objective and developed and published a document claiming 13 objectives for achieving the principal goal named “National Document for the Prevention and Control of Non-communicable Diseases and Related Risk Factors” [19]. The challenges of implementing the package of essential disease interventions in the Iranian health system were examined in 2015. The study reported that the reconstruction of the health system and encouraging cooperation between different governmental and non-governmental sectors and promoting health education could promote non-communicable disease intervention delivery [20]. Therefore, this study intended to use the scenario method to develop futures cardiovascular disease-related scenarios to illustrate possible situations, aiming to help policymakers make prompt or provident decisions to prevent increased myocardial infarction risk and provide country-specific policy options related to different scenarios.

## Methods

A comprehensive search was conducted to find relevant studies in MEDLINE, Scopus, Web of Science, and Google Scholar between 2002 and 2018. Besides, a manual search of these articles’ reference lists and bibliographies was executed to capture additional articles for consideration. The following keywords were used in all databases: “Key Factors,” “Strategy,” “Driver,” “Driving Force,” “Restraining Force,” “cardiovascular diseases,” “Heart Attack,” “Noncommunicable Diseases,” “Myocardial Infarctions,” “Control,” and “Prevention.”

Fifteen experts from the fields of management policy and health economics [7], community and preventive medicine [2], epidemiology [3], and cardiology [3] participated in completing and promoting the list of identified drivers. The STEEP framework (Social, Economical, Political, Environmental and Technological) was used to analyze the items of the final list.

**Fig. 1:**
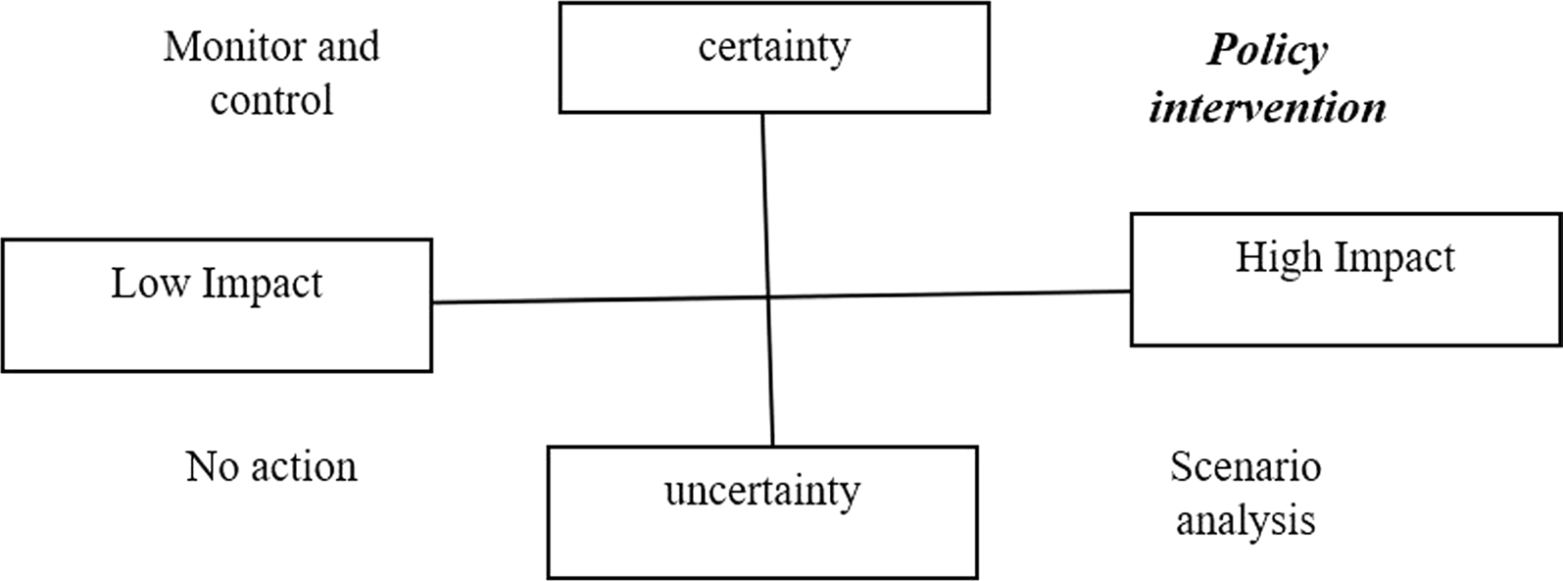
The identification of key uncertainties [21]

In the next step, we selected 18 pre-identified and well-known experts from all over the country and invited them to participate in the study via email, Skype calls, or in person. The purposeful sampling method was used, and experts with the most knowledge and experience in the prevention of CVD were selected. Then, using the final list of drivers, we prepared a questionnaire (Q1), sent them to the experts, and asked them to grade the drivers regarding their degree of importance and the degree of uncertainty in the range of 1 to 10. Admittedly, in this grading system, 1 indicates the driver with the lowest impact or the most uncertain one that might happen in futures and 10 indicates the highest impact that the driver could have regarding the cardiovascular event or the most certain driver that could influence the event. (Additional file 1). After that, we plotted all of them in a two-dimensional matrix to extract the set of drivers with the highest impact and certainty (policy intervention), using their mean scores of impacts and uncertainty (Fig. 1).

In order to draw a cross-impact matrix, we made a second questionnaire (Q2) which entailed three groups of drivers categorized upon their similarities. Then, we asked the experts to assess the drivers’ one-to-one relationships as positive or negative influences relative to each other and determine the magnitude of the influence by giving scores ranging from − 3 to + 3. Finally, the Scenario Wizard software 4.31 was used to perform a cross-impact matrix balance analysis, in which the inconsistency coefficient was set on 2. Also, total impact score was used.

In the next step, we listed the most believable scenarios that were returned running Scenario Wizard software and illustrated a causal network determining the interaction of the drivers with the final situation in the scenario. After that, based on the illustrations, we narrated a most relatable story for each scenario and reviewed them and edited them several times. In order to determine the validity of the scenarios, we developed a third questionnaire (Q3) via which the experts were asked to grade the strength of the scenarios answering the following questions: “Is Believable?”, “Is it challenging?”, “Is it internally consistent?”, “Is it relevant?”, and “is it well structured?”. The grading scale for determining the strength of each scenario was in the range of 1–10. The mean score ≥ 5 was considered acceptable in terms of the validity of the scenario.

In the next step, utilizing a fourth questionnaire (Q4), the experts were asked to select three of the all valid scenarios as “the most optimistic scenario,” “the most pessimistic scenario,” and “the most probable scenario.” Experts could assign “the most probable scenario” to the same scenario that might be determined as the most optimistic or the most pessimistic scenario. Additionally, they were asked to suggest and list strategic approaches to address the alleged scenarios if any of them might occur in the futures. After that, we used the analytical hierarchy process (AHP) to rank preferences of the experts for alternative approaches and weight their preferences according to four attributes listed as “effectiveness” in terms of increasing the overall population health, “political feasibility,” “economically beneficial,” and the “required budget” for implementing the proposed approach. Expert Choice 11.5 was used for AHP analysis.

## Results

**Fig. 2:**
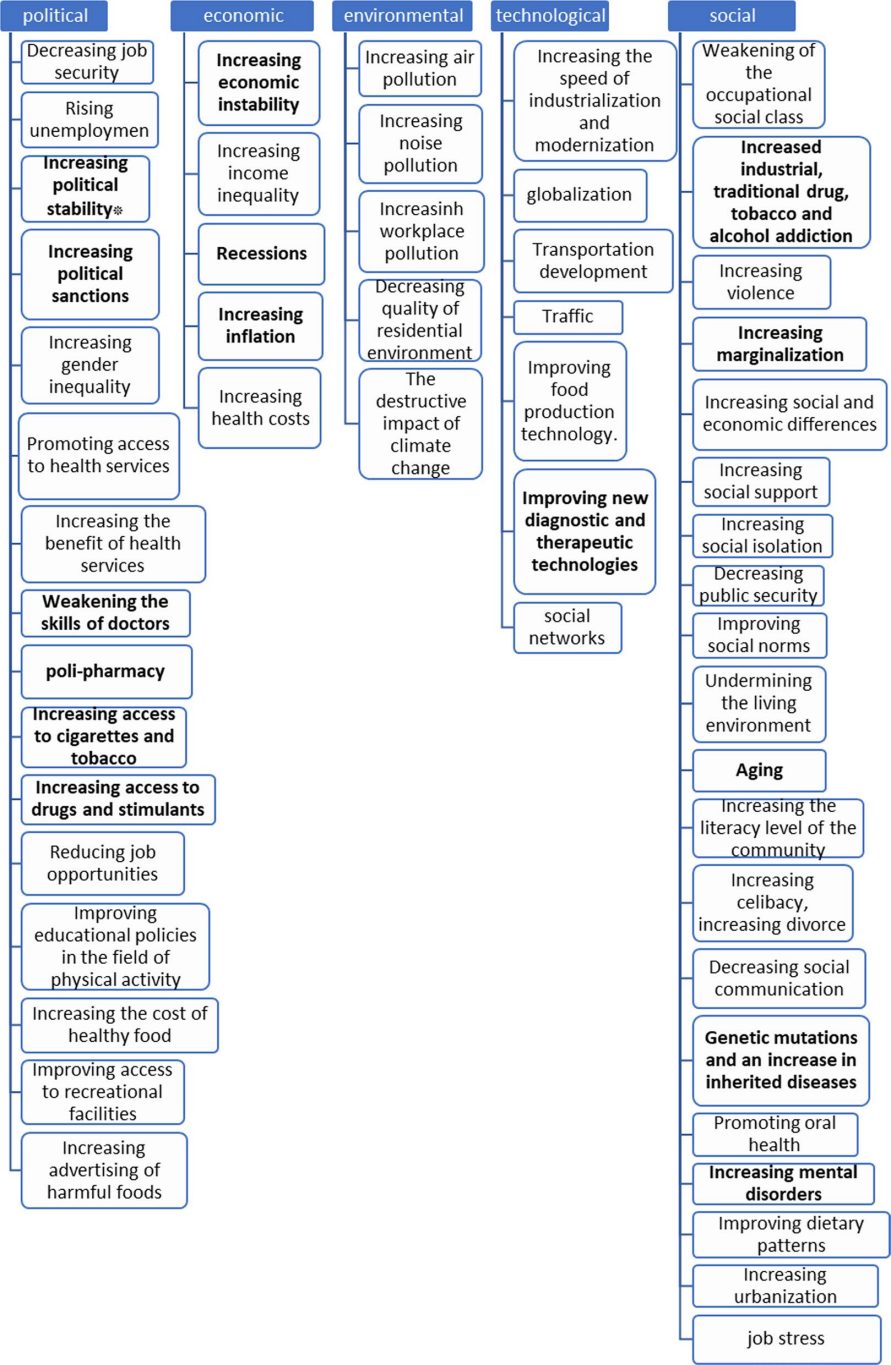
Drivers identified in STEEP framework

The initial search resulted in 5332 articles in four databases. Then, after cleaning for duplicates and articles without full text, 274 original articles from the remaining 1852 were screened using titles and abstracts. The papers that met all the eligibility criteria were selected for deliberate review. After reading full texts, 64 articles were included as the prime source for identifying the drivers. Preliminary, 98 key drivers were identified from included studies; then, after eliminating duplicates and similar factors, 38 items were listed as key drivers, which then promoted to a 53-item list adding the suggested drivers by the experts (Fig. 2).

**Fig. 3:**
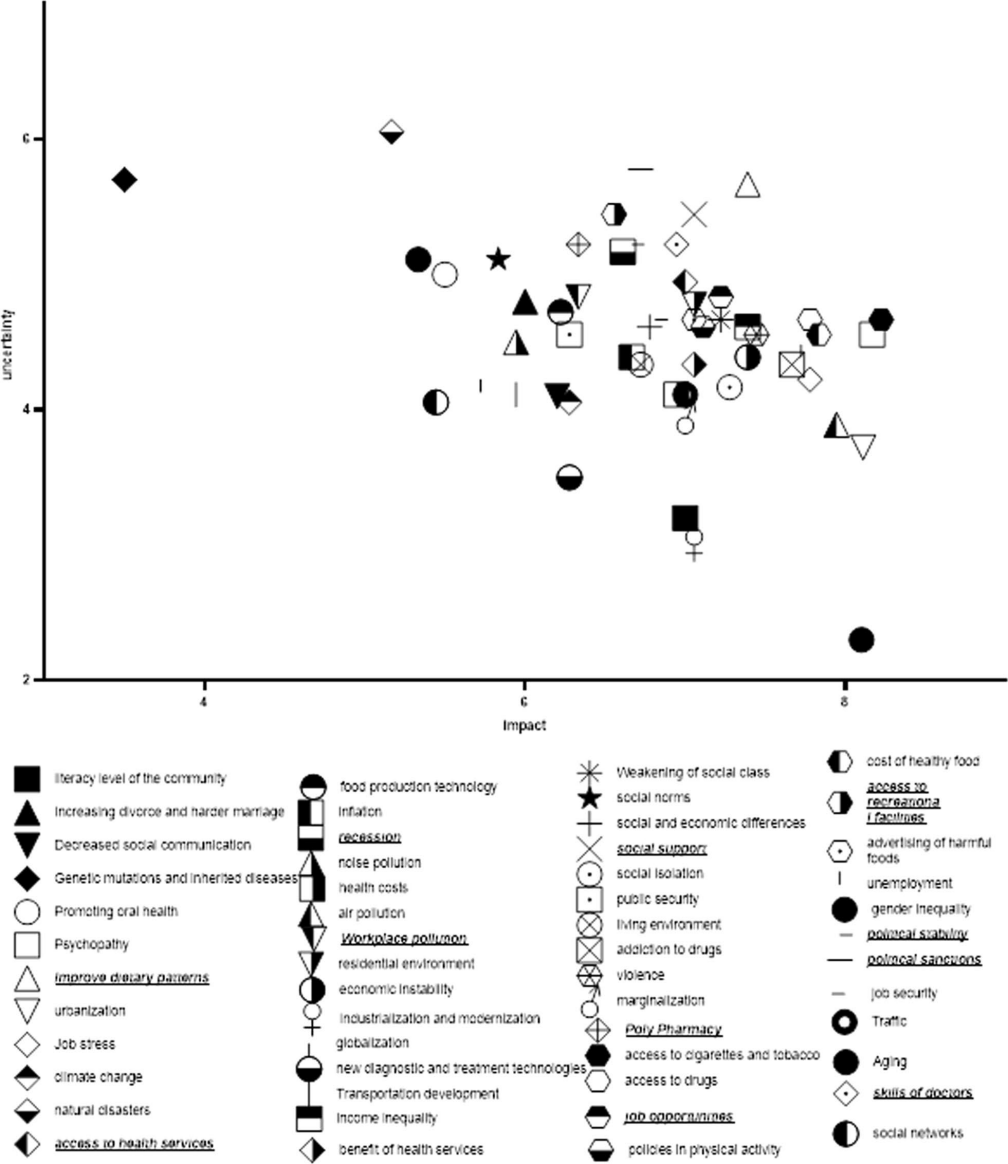
The identification of key uncertainties

The dissemination of the factors determined by their average scores for impactfulness and uncertainty is demonstrated in Fig. 3. According to the plot, 11 items were determined as most certain and the most impactful drivers: 1. improved diet quality, 2. increased social support, 3. increase in diplomatic sanctions, 4. the inadequacy of skilled physicians, 5. improved health services accessibility, 6. a decline in the numbers of job opportunities, 7. improved political stability, 8. increased accessibility to recreational facilities, 9. increased polypharmacy, 10. economic depression, and 11. increased workplace pollutant exposures.

**Fig. 4:**
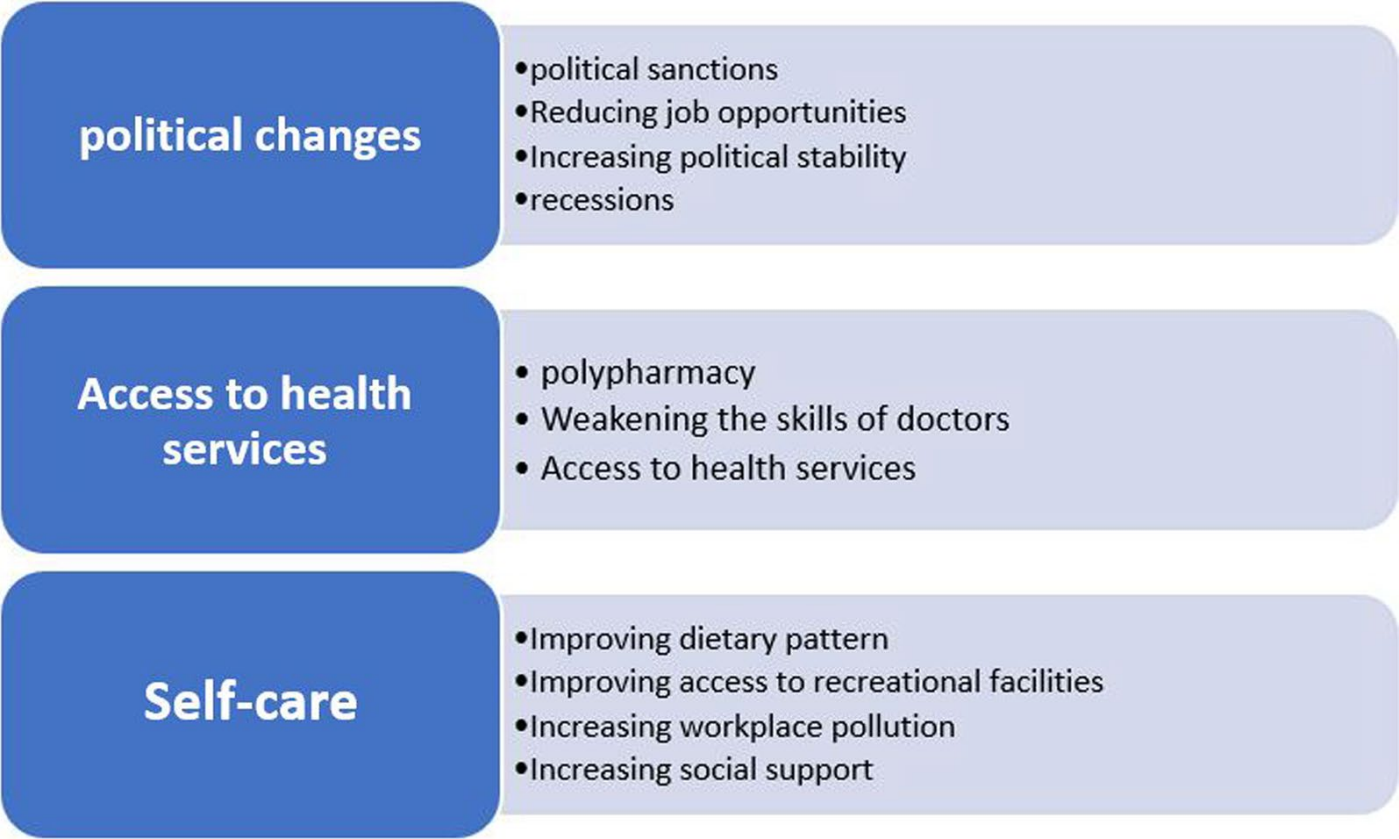
Factors with high uncertainty and high impact potential

The cross-impact matrix entailed all the eleven selected drivers classified into three groups based on their conceptual and semantic similarities, i.e., “political evolutions” (items 1 to 4), “health services accessibility” (items 5 to 7), and “self-care” (items 8 to 11) (Fig. 4).

The cross-impact matrix balance analysis on one-to-one relationships of the data returned six scenarios, and subsequently, each received a careful and deliberate narration. From all the validation questionnaires (Q3) sent to the experts, 16 (88.9%) were sent back. Table 1 summarizes the final validation results of the narrations in which the narrations entitled “Bird in a cage” and “Hope and Fear” received the highest validity scores.

**Table 1:** Consistent and believable scenarios

Criteria	Scenario 1*	Scenario 2	Scenario 3	Scenario 4	Scenario 5*	Scenario 6*
Believable	5.68	5.9	6.03	5.80	7.40	7.59
challenging	6.83	6.8	6.78	6.95	7.39	7.49
Internal compatibility	7.24	7.07	6.96	6.81	7.52	7.5
Relevant	6.35	6.26	6.33	6.18	6.81	6.76
Design	7.96	6.85	7.74	7.18	7.71	7.21
Total	6.81	6.57	6.76	6.58	7.36	7.31
Acceptable criteria	> 5	> 5	> 5	> 5	> 5	> 5

*Selective scenarios were underlined

The results of the fourth questionnaire (Q4), which was designed to determine “the most optimistic scenario,” “the most pessimistic scenario,” and “the most probable scenario,” revealed that the experts agreed upon the scenario (Table 2).

**Table 2:** Policy options for probabilistic, optimistic, and pessimistic scenarios based on priority

Scenario	Policy options based on priority
“A gentle path” Optimistic	Improving the infrastructure for providing prevention services
	Design and regenerate infrastructure and programs to strengthen preventive prevention to reduce risk factors for cardiovascular disease in the community
	Using the power of the private and non-governmental sector to provide cardiovascular disease prevention care on the outskirts of cities and slums
	Utilizing health marketing methods and strategies in the prevention of cardiovascular disease
“Hope and Fear” Pessimistic	Strengthen health-related restrictive and punitive legislation
	Empowering the community in order to improve the business situation and health
	Improving the infrastructure for providing prevention services
	Modeling social prescription in the prevention of cardiovascular disease
“Bird in a cage” Possible	The use of NGOs and charities in the prevention of cardiovascular disease
	Strengthen health-related restrictive and punitive legislation
	Empowering the community in order to improve the business situation and health
	Improving the infrastructure for providing prevention services

## Discussion

In this study, we examined cardiovascular precipitating drivers, within which 53 divers identified as influential in the occurrence of cardiovascular diseases. Overall, childhood and development circumstances, the quality of places where people reside, and social support seem to have immense importance among the attributing drivers. Hence, the prevalence, incidence, and quality of life of those affected with cardiovascular diseases are impacted by all the functions of health systems, and the exposure to the CVD’s risk factors changes with the governmental and sociocultural circumstances, all policies regarding promoting physical activity, developing recreational facilities, transportation, preserving and expanding urban green spaces, residential and workplace environments, and tobacco control influences the burden of the cardiovascular diseases in the communities[22].

Therefore, the interventions targeting the living conditions of the community members and urban designs aiming to reduce risk factors of CVDs should not be overlooked in designing and implementing preventing and controlling measures and making policies [23–29].

The application of futures studies in health policy researches enables policymakers to have a potentially holistic perspective view over future health outcomes. In this regard, Goharinezhad et al. stated that despite the aging population of Iran, the government do not prioritize building infrastructure for future health care coverage; therefore, the different scenarios illustrating futures of the quality of health services for the elderly and health outcomes would give them a chance to act wisely and give a better chance to the optimistic scenario to manifest [30]. Result of study in England was shown that coronary heart disease mortality reductions of up to 45%, accompanied by significant reductions in area deprivation mortality disparities, would be possible by implementing optimal preventive policies [31].

Various studies aimed to depict the futures of health conditions in Iran and risk factor reduction policies identified similar uncertainties for raising the probability of unfolding an optimistic scenario. Some of these uncertainties are political ideation overarching decision-making in the country, overall economy, environmental circumstances such as pollution or transportation, availability, and accessibility of quality health care and sufficient self-management within the population [29, 30, 32]. These uncertainties also composed the substantial basis of our scenarios, indicating that policymakers should pay deliberate attention to challenges that these items could bring about in the futures.

Like most upper-middle-income countries, Iran struggles with the burden of non-communicable diseases and has committed to achieving a 25% reduction in premature mortality from NCDs by 2025 (current WHO target) [33]. Having an unstable economy, the health system and people need the philanthropic and targeted activities of the NGOs in addition to the governmental health-related actions. Therefore, a large part of the policies and action plans of these organizations should be aligned with the health-related objectives and risk reduction behaviors such as increasing health literacy and physical activity opportunities, adopting and implementing policies for ensuring the availability and accessibility of healthy and balanced diet and promoting other preventive services [34, 35]. Admittedly, the government should allocate adequate resources for research, education, and promoting preventive measures to increase the effectiveness of interventions targeting CVDs, collaborate with other sectors, and encourage population participation in health care decision-making. Supposedly, with the optimistic scenario in hand, such goals are achievable; however, futures Iran is far from fulfilling WHO’s 2025 targets in the pessimistic and probable scenarios.

The project FRESHER, whose objective was to represent alternative futures where the detection of emerging health scenarios is used to test futures research policies to tackle the burden of NCDs, deified four scenarios which overall were narrated based on various sociocultural, technological, economic, and environmental factors. The authors discussed that positive depictions could only be realized in a society where the resources are spread equitably, sustainable growth does not depend only on limited resources, and health care is more person-centered and community-based care, people age in a fruitful process, and in this urbanized societies, people comply with healthy lifestyles and engage in life-long learning opportunities and have sufficient health literacy [36]. As is stated, our scenarios have also emphasized socio-demographic trends, economic factors, and technology advancements in depicting a future with decreased CVD incidents. However, in the circumstances with an unstable economy, allegedly, any political changes could deviate each optimistic or pessimistic scenario from its original narrative; moreover, costly technology-based health services, which still imposes significant concerns to low- and middle-income countries, would paradoxically jeopardize equitable health care delivery. Zahedi et al. conducted a study aiming to forecast the role of Non-Governmental Organizations (NGOs) in the futures of the health system’s accountability in Iran. Similar to the scenarios portrayed in our study, empowering the communities to take hold of their health, encouraging community participation, and empowering the NGO’s to expand their activities are the main principles that would increase accountable health care delivery in futures Iran [34].

## Limitations

In futures studies, the determination of the uncertainties and composing scenarios depend on the logic and intuitions of those who participate as experts. Although we tried to use a comprehensive roster of experts, given that futures studies are new in our country and have the experts relatively unfamiliar with its methodology, we had difficulty finding experts to assess and speculate Iran’s futures of CVDs in the framework of futures studies. Furthermore, due to limitations that the COVID-19 pandemic wreaked, some of the supposed panels of experts were canceled, and the experts’ opinions were asked via email. Likewise, we could not assess the objectivity of the suggested policies and wrote our report based on the array of opinions collected from the experts.

## Conclusion

The results of this study have shed light on how futures of cardiovascular diseases in Iran might be in the upcoming years and by depicting influential drivers and connecting them to the CVD outcomes. Furthermore, these findings demonstrate that futures of CVDs in Iran strongly depend on social and economic factors that any reforms or alteration of policies or regulations could substantially change regarding the alleged scenarios. These insights would help policymakers to intentionally watch the impacting trends and attributing factors predisposing populations to burden CVDs, among which fostering healthy behaviors that are directly influenced by economic and political circumstances.

The optimistic scenario illustrates improving social and economic circumstances. In this scenario, increased quality care delivery and popularity of self-management within the communities decreases the burden of CVD events. Priorities are set on rectifying infrastructures for increasing prevention services and focusing on decreasing population attributable risk factors. This scenario seems to be promising concerning the fulfillment of the National non-communicable disease document’s objectives.

The pessimistic and the probable scenarios do not foresee any improvement in social and economic circumstances in futures and predict a rise in cardiovascular events. These scenarios encourage policymakers to adopt restrictive health policies and give greater scope to NGOs and philanthropists to promote preventive health measures in the communities. If these scenarios take place in futures, the burden of CVDs would be extensive. Its prevention depends on the government and the nation, requiring them to align their goals with increasing health literacy, promoting environmental conditions, and complying with a healthy lifestyle.

## Supplementary Information

**Additional file 1.** Experts’ characteristics

## Data Availability

The datasets used and/or analyzed during the current study are available from the corresponding author on reasonable request.
