# Supplementary material for: Future of myocardial infarction mortality in Iran: a scenario-based study
Source: J Health Popul Nutr. 2023 Mar 16;42:19. doi: 10.1186/s41043-023-00356-8 (PMC10018627; doi:10.1186/s41043-023-00356-8)
Supplement: Supplementary file 1 — Additional file 1. Experts’ characteristics [file 41043_2023_356_MOESM1_ESM.docx]

**Supplementary 1. Experts’ characteristics**

| **Participants in the selection three of scenarios** | **Participants in the validity of the scenario** | **Participants in the determination scores of Cross Impact matrix balance analysis** | **Participants in the determination degree of importance and the degree of uncertainty** | **Participants in finalizing items** | **Field of expertise** | **Gender** | **Expert** |
| --- | --- | --- | --- | --- | --- | --- | --- |
| * | * | * | * | * | Health policy | M | 1 |
| * | * | * | * | * | Health service management | M | 2 |
| * | * | * | * | * | Health economics | F | 3 |
| * | * | * | * | * | Cardiology | M | 4 |
| * | * | * | * | * | Epidemiology | F | 5 |
| * | * | * | * | * | Cardiology | F | 6 |
| * | * | * | * | * | Social medicine | M | 7 |
| * | * | * | * | * | Cardiology | M | 8 |
| * | * | * | * | * | Social medicine | F | 9 |
| * | * | * | * | * | Health policy | F | 10 |
| * | * | * | * | * | Epidemiology | F | 11 |
| * | * | * | * | * | Epidemiology | M | 12 |
| * | * | * | * | * | Social medicine | M | 13 |
| * | * | - | * | * | Health service management | M | 14 |
| * | - | * | * | * | Health service management | F | 15 |
| - | - | * | * | - | Health economics | M | 16 |
| - | - | - | * | - | Social medicine | F | 17 |
| - | * | - | * | - | Cardiology | M | 18 |
